# COPD Underdiagnosis and Misdiagnosis in a High-Risk Primary Care Population in Four Latin American Countries. A Key to Enhance Disease Diagnosis: The PUMA Study

**DOI:** 10.1371/journal.pone.0152266

**Published:** 2016-04-13

**Authors:** Alejandro Casas Herrera, Maria Montes de Oca, Maria Victorina López Varela, Carlos Aguirre, Eduardo Schiavi, José R. Jardim, PUMA Team

**Affiliations:** 1 Fundación Neumológica Colombiana, Escuela de Medicina y Ciencias de la Salud, Universidad del Rosario, Bogotá, Colombia; 2 Servicio de Neumonología, Hospital Universitario de Caracas, Facultad de Medicina, Los Chaguaramos, 1030, Universidad Central de Venezuela, Caracas, Venezuela; 3 Universidad de la República, Facultad de Medicina, Universidad de la República, Hospital Maciel, Montevideo, Uruguay; 4 Fundación Neumológica Colombiana, Bogotá, Colombia; 5 Hospital de Rehabilitación Respiratoria María Ferrer, Finochietto 849, Buenos Aires, Argentina; 6 Escola Paulista de Medicina at Federal University of São Paulo, São Paulo, Brazil; Lee Kong Chian School of Medicine, SINGAPORE

## Abstract

**Background:**

Acknowledgement of COPD underdiagnosis and misdiagnosis in primary care can contribute to improved disease diagnosis. PUMA is an international primary care study in Argentina, Colombia, Venezuela and Uruguay.

**Objectives:**

To assess COPD underdiagnosis and misdiagnosis in primary care and identify factors associated with COPD underdiagnosis in this setting.

**Methods:**

COPD was defined as post-bronchodilator (post-BD) forced expiratory volume in 1 second/forced vital capacity (FEV_1_/FVC) <0.70 and the lower limit of normal (LLN). Prior diagnosis was self-reported physician diagnosis of emphysema, chronic bronchitis, or COPD. Those patients with spirometric COPD were considered to have correct prior diagnosis, while those without spirometric criteria had misdiagnosis. Individuals with spirometric criteria without previous diagnosis were considered as underdiagnosed.

**Results:**

1,743 patients were interviewed, 1,540 completed spirometry, 309 (post-BD FEV_1_/FVC <0.70) and 226 (LLN) had COPD. Underdiagnosis using post-BD FEV_1_/FVC <0.70 was 77% and 73% by LLN. Overall, 102 patients had a prior COPD diagnosis, 71/102 patients (69.6%) had a prior correct diagnosis and 31/102 (30.4%) had a misdiagnosis defined by post-BD FEV_1_/FVC ≥0.70. Underdiagnosis was associated with higher body mass index (≥30 kg/m^2^), milder airway obstruction (GOLD I–II), black skin color, absence of dyspnea, wheezing, no history of exacerbations or hospitalizations in the past-year. Those not visiting a doctor in the last year or only visiting a GP had more risk of underdiagnosis. COPD underdiagnosis (65.8%) and misdiagnosis (26.4%) were less prevalent in those with previous spirometry.

**Conclusions:**

COPD underdiagnosis is a major problem in primary care. Availability of spirometry should be a priority in this setting.

## Introduction

Chronic obstructive pulmonary disease (COPD) is a considerable cause of morbidity and mortality. Sustained disease prevalence is partly due to the increasing age of the world’s population, the continued smoking of tobacco and in low-income countries to biomass exposure. Knowledge of the burden of disease in all settings is important for health systems worldwide.

Recently, an analysis of national and international surveys of general populations has shown substantial heterogeneity in COPD prevalence rates and high levels of COPD underdiagnosis [1]. In Latin America, the PLATINO study (Latin American Project for Investigation of Obstructive Lung Disease) showed a COPD prevalence of 14.3% using a ratio of the post-bronchodilator (post-BD) forced expiratory volume in 1 second/forced vital capacity (FEV_1_/FVC) <0.70 (fixed ratio) to define COPD [2], and a prevalence of underdiagnosis of 12.7% (COPD patients without a previous diagnosis; this ranged from 6.9% in Mexico City to 18.2% in Montevideo) [3]. In addition, a high proportion of subjects without prior COPD diagnosis (89%) were reported. The main factors associated with COPD underdiagnosis were: younger age, lower severity of airway obstruction and fewer respiratory symptoms [3]. Another subanalysis of the PLATINO study showed a lower prevalence of COPD using the definitions based on the lower limit of normal (below the 5th percentile) of the FEV_1_/FVC, especially among the elderly in comparison with fixed ratio [4].

Data on underdiagnosis in primary care are limited [5–7]. Acknowledgement of the problem in this setting is essential for the planning and implementation of strategies for the detection, accurate diagnosis and early treatment of the disease.

To the authors’ knowledge, there is no any international multicenter study with adequate sample size that has assessed COPD underdiagnosis and misdiagnosis in the primary care setting. The present study aims to assess the magnitude of the problem of underdiagnosis and misdiagnosis in a primary care population at high risk of COPD in four Latin American countries, and to identify the possible factors associated with COPD underdiagnosis in this setting.

## Materials and Methods

The PUMA study (Prevalence Study and Regular Practice, Diagnosis and Treatment, Among General Practitioners in Populations at Risk of COPD in Latin America) was conducted in the primary care setting of four Latin American countries: Argentina, Colombia, Venezuela and Uruguay (Clinical Trial Registration: NCT01493544). Complete details of the methodology have been published previously [8]. Briefly, the PUMA study is a multicenter, multinational, cross-sectional, non-interventional study. Participating sites included primary care centers (family doctors, general practitioners, etc.) with no direct connection to respiratory medicine specialists. These sites were selected to reflect the reality of national primary care practice in terms of geographical distribution (urban or rural) and healthcare sector (public or private). The ethics committees for each site involved in the study approved the protocol and all participants gave their written informed consent.

All patients ≥40 years of age and at risk for COPD (current or former smokers, and/or history of exposure to biomass, mainly wood smoke) attending primary care settings for any reason were included in the study. Patients were recruited during spontaneous routine or scheduled visits in which their medical appointment was unrelated to the study (with or without respiratory symptoms). Participants completed the PUMA study questionnaire (a modified version of the PLATINO study questionnaire), which was overseen by trained staff and included information on the following factors: demography, smoking habits, education, employment, respiratory symptoms, respiratory diagnoses, use of respiratory medication, and prior spirometric testing. A copy of the questionnaire is available online with the supplementary material. Data on prior medical diagnosis of tuberculosis, asthma, chronic bronchitis, emphysema, COPD, self-reported exacerbations (determined in response to the following question: In the last 12 months, have you had an acute change in your usual respiratory symptoms (shortness of breath and/or cough and/or phlegm) out of the daily variability, which has led to changing your regular medication? [Yes/No] a. How many such episodes have you had in the past 12 months? b. For how many of these episodes did you need to see a doctor in the past 12 months? c. For how many of these episodes were you hospitalized in the past 12 months?) and hospitalizations were also obtained.

Spirometry was undertaken using a portable, battery-operated ultrasonic spirometer (Easy One; ndd Medical Technologies, Zurich, Switzerland). Spirometry tests were performed at baseline and 15 min after the administration of 400 μg salbutamol, according to the American Thoracic Society criteria of acceptability and reproducibility.

### Definitions

1. Two definitions of COPD were used: first, a ratio of the post-bronchodilator (post-BD) FEV_1_/FVC <0.70 (fixed ratio), and second, the same ratio under the lower limit of normality (LLN; defined as the lower 5^th^ percentile for predicted post-BD FEV_1_/FVC). The reference equations used to calculate the LLN were the PLATINO study reference equations [9,10]. Severity of COPD airway obstruction was graded using the Global Initiative for Chronic Obstructive Lung Disease (GOLD) criteria [11].
2. Prior diagnostic label of COPD was determined using a self-reported physician diagnosis of emphysema, chronic bronchitis, or COPD.
3. Those individuals with previous medical diagnosis and spirometric criteria of COPD were considered as correct prior medical diagnosis.
4. Among those with previous medical diagnosis and not spirometric criteria of COPD, the misdiagnosis or incorrect prior medical diagnosis was attributed.
5. Underdiagnosis of COPD was considered when participants had spirometric criteria of COPD (post-BD FEV_1_/FVC <0.70 or <LLN), but had no previous medical diagnosis of COPD.

### Statistical Analysis

Descriptive analyzes were performed using absolute and relative frequencies for categorical variables. Some descriptions were performed with total sample and country stratified. In order to establish the associated factors for underdiagnosis, crude and adjusted Poisson regression models were performed since the outcome is dichotomous and the study design is cross-sectional. On the adjusted model, all variables in the table were used together. Analysis was adjusted for all variables in the tables: sex, age, skin color, schooling, BMI, dust, wheezing, cough, sputum, comorbidity score, exacerbations and hospitalizations for exacerbation. All analyzes were performed using statistical package Stata, version 13.1 (Statcorp, College Station, Texas, TX, USA).

## Results

Detailed descriptions of participation rates have been published previously [7]. In summary, 1,743 individuals completed the interviews and 1,540 performed acceptable spirometry. Among them there were 309 (20.1%) and 226 (14.7%) patients with COPD defined by post-BD FEV_1_/FVC <0.70 and LLN, respectively. From the 309 patients with COPD by post-BD FEV_1_/FVC <0.70, 90 patients (29.1%) do not have COPD by the LLN.

**Fig 1:**
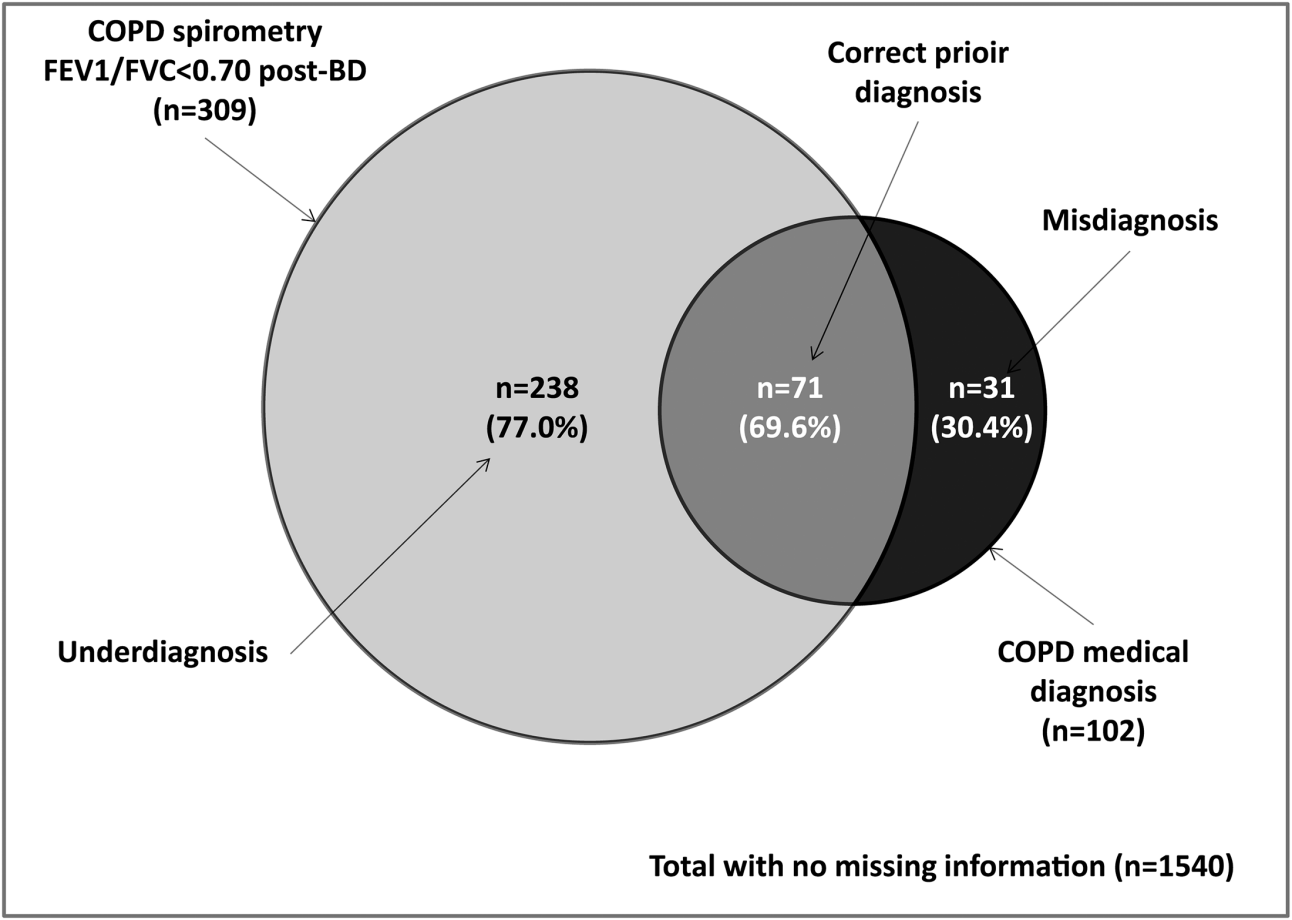
Proportion of underdiagnosis among those patients with spirometric diagnosis of COPD by post-BD FEV_1_/FVC <0.70, and correct prior diagnosis or misdiagnosis among those patients with previous COPD medical diagnosis.

The proportion of COPD underdiagnosis among those patients with spirometric diagnosis (post-BD FEV_1_/FVC<0.70 criteria), and the proportion of correct prior diagnosis and misdiagnosis among those patients with prior medical diagnosis of COPD by post-BD FEV_1_/FVC <0.70 criteria are presented in Fig 1. There were 102 patients with a prior medical diagnosis of emphysema, chronic bronchitis, or COPD. Among them, 71 patients (69.6%) had post-BD FEV_1_/FVC <0.70 (correct prior diagnosis), while 31 patients (30.4%) had post-BD FEV_1_/FVC ≥0.70 (misdiagnosis). From the 309 COPD subjects by post-BD FEV_1_/FVC <0.70 criteria 82 subjects (26.5%) have prior asthma medical diagnosis. This probably represents asthma misdiagnosis.

**Fig 2:**
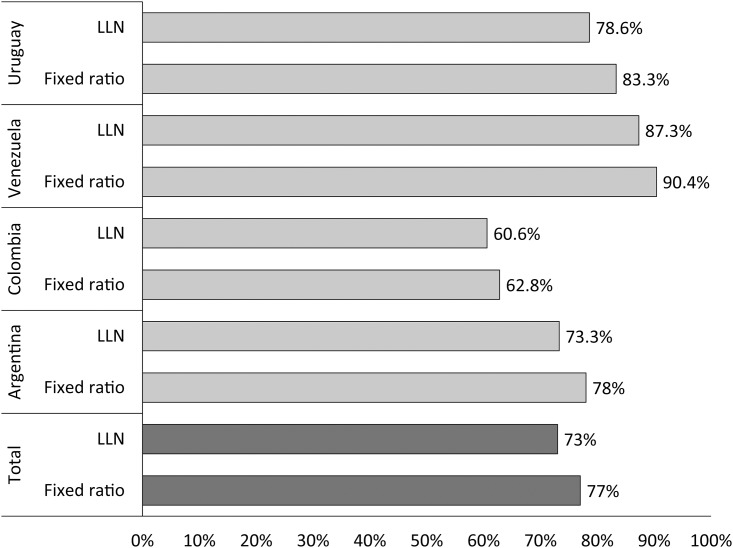
Proportion of COPD underdiagnosis according to different criteria (post-BD FEV_1_/FVC <0.70 and LLN), total and by country. P-values for the comparison of underdiagnosis by Fixed ratio and the LLN criteria: Uruguay p = 0.73; Venezuela p = 0.57; Colombia p = 0.78; Argentina p = 0.42; Total p = 0.33.

**Fig 3:**
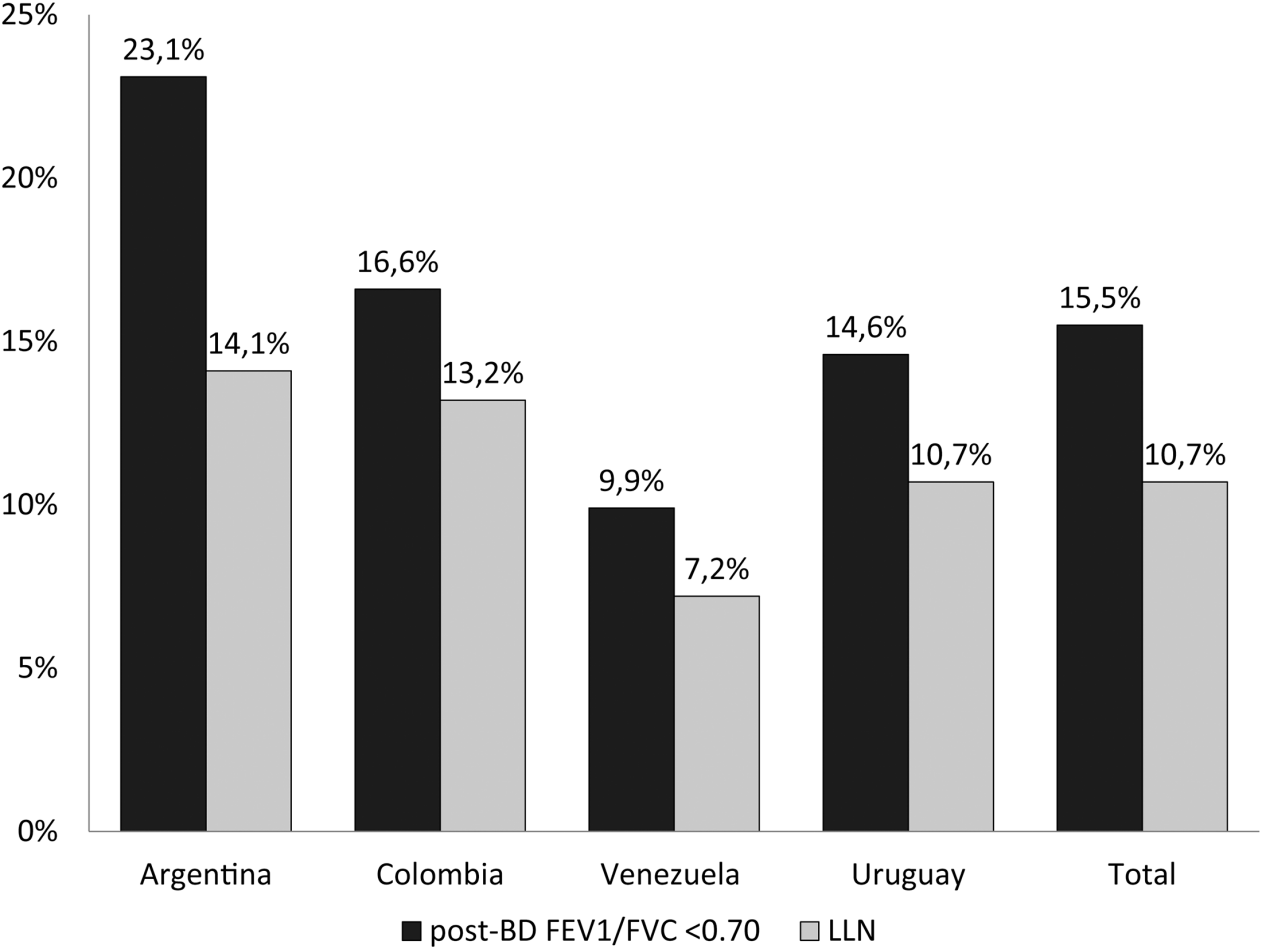
Population prevalence of underdiagnosis among those patients with spirometry criteria of COPD: A) post-BD FEV_1_/FVC <0.70; and B) LLN.

The proportion of COPD underdiagnosis (according to the different criteria) overall and by country is shown in Fig 2. The overall COPD underdiagnosis using the post-BD FEV_1_/FVC <0.70 definition was 77% (ranging from 63% in Colombia to 90% in Venezuela), and using LLN was 73% (ranging from 61% in Colombia to 87% in Venezuela) (Fig 2). The population prevalence of COPD underdiagnosis (according to both criteria), overall and by country, is shown in Fig 3. The overall COPD underdiagnosis prevalence using the post-BD FEV_1_/FVC <0.70 definition was 15.5% (238/1540 patients) and using the LLN definition was 10.7% (165/1540 patients) (Fig 3).

Description of the population’s characteristics according to selected variables, prevalence of COPD (post-BD FEV_1_/FVC <0.70 and LLN criteria), patients with correct prior medical diagnosis, and the underdiagnosis proportion (post-BD FEV_1_/FVC <0.70 and LLN criteria) are shown in Table 1. The COPD underdiagnosis was higher among those patients that were black, younger age (40–49 years), had a higher body mass index (BMI; ≥30 kg/m^2^), without wheezing, dyspnea and history of exacerbations or hospitalizations due to exacerbations in the past year (Table 1). On the other hand, prior medical diagnosis was more frequent among those patients that were older (≥60 years), had a lower BMI (<25 kg/m^2^), had more severe airway obstruction (GOLD III–IV), had respiratory symptoms, and history of exacerbations or hospitalizations due to exacerbations in the past year. Table 2 shows results from logistic regression modeling to identify factors associated to COPD underdiagnosis according to the different criteria (FEV_1_/FVC <0.70 and LLN). The crude odds of COPD underdiagnosis were significantly higher among individuals who were black, had a higher BMI (≥30 kg/m^2^), without dyspnea and wheezing in past year, had mild to moderate airway obstruction (GOLD I–II) and no history of exacerbations and hospitalizations due to exacerbations in the past year, whereas the adjusted odds of COPD underdiagnosis were significantly higher only in those individuals who were black, had a higher BMI (≥30 kg/m^2^), and had mild to moderate airway obstruction (GOLD I–II).

**Table 1:** Sample description according to selected variables, prevalence of COPD (medical diagnosis, post-BD FEV_1_/FVC <0.70 and LLN criteria) and underdiagnosis proportion of COPD (fixed ratio and LLN criteria).

			COPD (FEV_1_/FVC<0.70 post-BD) N = 309		COPD (LLN) N = 226	
Variable	N (%)	Prevalence of prior medical diagnosis of COPD (%)	Prevalence of COPD (%)	Underdiagnosis (%)	Prevalence of COPD (%)	Underdiagnosis (%)
Sex						
Female	876 (50.3)	5.7	17.3	77.2	13.0	73.5
Male	867 (49.7)	7.5	22.9	76.9	16.4	72.6
Age (complete years)						
40–49	328 (18.8)	1.0	2.3	85.7	4.3	92.3
50–59	589 (33.8)	6.3	13.1	76.1	10.5	71.9
60+	826 (47.4)	9.3	33.1	77.1	22.3	71.8
Skin color						
White	882 (50.9)	5.5	19.7	79.5	13.7	76.5
Brown	749 (43.8)	8.7	21.7	72.7	16.7	67.6
Black	92 (5.3)	1.3	12.5	100.0	10.0	100.0
Schooling (complete years of formal education)						
0–8	892 (51.2)	9.3	21.7	75.2	16.0	69.7
9+	851 (48.8)	5.9	18.5	79.2	13.4	76.9
BMI (kg/m^2^)						
<25.0	514 (29.5)	10.8	31.2	73.9	25.1	68.5
25.0–29.9	682 (39.1)	5.6	17.7	74.8	12.2	73.0
≥30	547 (31.4)	4.1	13.0	87.5	8.3	85.4
Pack years smoked						
≤20	585 (34.6)	4.8	10.8	81.5	8.6	76.7
20–30	366 (21.6)	6.6	14.8	83.3	11.1	80.6
>30	740 (43.8)	10.7	29.8	74.2	21.5	70.6
Occupational exposure to dust						
No	956 (54.9)	5.3	18.3	79.4	13.0	75.5
Yes	786 (45.1)	8.2	22.2	74.7	16.7	70.7
Wheezing in past year						
No	1,489 (85.4)	5.2	17.9	81.7	12.8	77.5
Yes	254 (14.6)	15.2	33.0	62.2	25.4	59.6
Symptoms						
Dyspnea (yes)	756 (46.8)	10.7	27.7	68.7	22.0	65.3
Cough (yes)	571 (32.8)	9.5	27.2	75.6	19.9	69.7
Phlegm (yes)	523 (30.0)	11.5	30.3	71.9	22.4	67.0
Comorbidities score*						
0	520 (30.0)	4.2	15.7	79.7	12.3	79.3
1	663 (38.3)	8.1	20.9	74.2	16.2	69.8
2	391 (22.6)	6.9	24.5	80.2	16.6	72.7
3+	158 (9.1)	6.7	19.4	76.9	10.4	78.6
Exacerbations in past year						
No	1,619 (92.9)	5.1	19.2	81.9	13.6	79.1
Yes	123 (7.1)	28.4	32.4	36.4	29.4	33.3
Hospitalizations due to exacerbations in past year						
No	1,716 (98.5)	5.9	19.7	79.2	14.2	75.8
Yes	26 (1.5)	52.2	47.8	18.2	47.8	18.2
GOLD stages						
0	1,231 (79.9)	2.5	-	-	0.6	100.0
I	53 (3.4)	13.2	100.0	86.8	39.6	85.7
II	169 (11.0)	14.2	100.0	85.8	66.9	83.2
III-IV	87 (5.7)	46.0	100.0	54.0	97.7	54.1
**Total**	**1,743 (100.0)**	**6.6**	**20.1**	**77.0**	**14.7**	**73.0**

* Simple sum of the number of self-reported comorbidities, considering the following: heart diseases, hypertension, diabetes, lung cancer, stroke (or embolism or ischemia), tuberculosis and gastritis (or ulcer).

**Table 2:** Logistic regression models to identify factors associated to COPD underdiagnosis according to different criteria.

	COPD underdiagnosis (FEV_1_/FVC<0.70 post-BD)		COPD underdiagnosis (LLN)	
Variable	Crude PR (95% CI)	Adjusted PR (95% CI)	Crude PR (95% CI)	Adjusted PR (95% CI)
Sex	***P = 0*.*946***	***p = 0*.*383***	***P = 0*.*873***	***P = 0*.*553***
Female	1.00	1.00	1.00	1.00
Male	1.00 (0.88; 1.13)	1.06(0.93; 1.21)	0.99 (0.84; 1.16)	1.05 (0.89; 1.24)
Age (complete years)	***P = 0*.*775***	***P = 0*.*456***	***P = 0*.*023***	***P = 0*.*586***
60+	1.00	1.00	1.00	1.00
50–59	0.99 (0.85; 1.15)	0.92 (0.63; 1.34)	1.00 (0.83; 1.21)	0.90 (0.62; 1.29)
40–49	1.11 (0.82; 1.52)	0.94 (0.81; 1.10)	**1.29 (1.07; 1.55)**	0.97 (0.81; 1.16)
Skin color	***P<0*.*001***	***P = 0*.*057***	***P<0*.*001***	***P = 0*.*212***
White	1.00	1.00	1.00	1.00
Brown	0.91 (0.80; 1.04)	1.02 (0.90; 1.15)	0.88 (0.75; 1.05)	0.98 (0.83; 1.16)
Black	**1.26 (1.16; 1.36)**	**1.31 (1.05; 1.63)**	**1.31 (1.18; 1.45)**	**1.30 (0.97; 1.74)**
Schooling (complete years of formal education)	***P = 0*.*081***	***P = 0*.*575***	***P = 0*.*102***	***P = 0*.*480***
0–8	1.00	1.00	1.00	1.00
9+	1.05 (0.93; 1.19)	1.04 (0.91 1.18)	1.10 (0.94; 1.29)	1.06 (0.90; 1.26)
BMI (kg/m^2^)	***P = 0*.*025***	***P = 0*.*042***	***P = 0*.*047***	***P = 0*.*145***
<25.0	1.00	1.00	1.00	1.00
25.0–29.9	1.01 (0.87; 1.17)	0.96 (0.82; 1.13)	1.07 (0.88; 1.29)	1.00 (0.82; 1.23)
≥30	**1.18 (1.03; 1.36)**	**1.15 (1.00; 1.32)**	**1.25 (1.04; 1.49)**	**1.18 (0.99; 1.40)**
Pack years smoked	***P = 0*.*237***	***P = 0*.*320***	***P = 0*.*369***	***P = 0*.*805***
≤20	1.00	1.00	1.00	1.00
20–30	1.02 (0.85; 1.22)	0.99 (0.83; 1.17)	1.05 (0.83; 1.32)	1.01 (0.80; 1.29)
>30	0.91 (0.78; 1.06)	0.91 (0.79; 1.05)	0.92 (0.76; 1.12)	0.95 (0.79; 1.15)
Occupational exposure to dust	***P = 0*.*330***	***P = 0*.*721***	***P = 0*.*421***	***P = 0*.*830***
Yes	1.00	1.00	1.00	1.00
No	1.06 (094; 1.20)	0.98 (0.86; 1.11)	1.07 (0.91; 1.25)	0.98 (0.84; 1.15)
Wheezing in past year	***P = 0*.*004***	***P = 0*.*215***	***P = 0*.*0*.*25***	***P = 0*.*514***
Yes	1.00	1.00	1.00	1.00
No	**1.31 (1.09; 1.59)**	1.13 (0.93; 1.36)	**1.30 (1.03; 1.63)**	1.08 (0.86; 1.35)
Symptoms				
Dyspnea	***p<0*.*001***	***p = 0*.*207***	***p<0*.*001***	***p = 0*.*382***
Yes	**1.00**	1.00	**1.00**	1.00
No	**1.32 (1.17; 1.49)**	1.09 (0.96; 1.24)	**1.36 (1.17; 1.57)**	1.08 (0.91; 1.28)
Cough	***p = 0*.*593***	***p = 0*.*502***	***p = 0*.*331***	***p = 0*.*859***
Yes	1.00	1.00	1.00	1.00
No	1.03 (0.91; 1.17)	0.95(0.83; 1.09)	1.08 (0.92; 1.28)	0.98 (0.82; 1.18)
Phlegm	***p = 0*.*062***	***p = 0*.*322***	***p = 0*.*070***	***p = 0*.*428***
Yes	1.00	1.00	1.00	1.00
No	1.13 (0.99; 1.28)	1.10 (0.94; 1.29)	1.17 (0.99; 1.37)	1.08 (0.89; 1.31)
Comorbidities score*	***P = 0*.*719***	***P = 0*.*135***	***P = 0*.*565***	***P = 0*.*442***
0	1.00	1.00	1.00	1.00
1	0.93 (0.80; 1.09)	0.96 (0.81; 1.13)	0.88 (0.73; 1.06)	0.93 (0.76; 1.13)
2	1.01 (0.86; 1.18)	1.10 (0.94; 1.28)	0.92 (0.74; 1.13)	1.05 (0.85; 1.28)
3+	0.96 (0.76; 1.23)	0.93 (0.71; 1.21)	0.99 (0.73; 1.34)	0.98 (0.70; 1.37)
Exacerbations in past year	***P<0*.*001***	***P = 0*.*060***	***P<0*.*001***	***P = 0*.*805***
Yes	1.00	1.00	1.00	1.00
No	**2.25 (1.43; 3.55)**	**1.56 (0.98; 2.47)**	**2.37 (1.42; 3.96)**	1.56 (0.94; 2.59)
Hospitalizations due to exacerbations in past year	***P = 0*.*022***	***P = 0*.*222***	***P = 0*.*026***	***P = 0*.*249***
Yes	1.00	1.00	1.00	1.00
No	**4.36 (1.24; 15.31)**	2.32 (0.60; 8.99)	**4.17 (1.18; 14.68)**	2.24 (0.572; 8.88)
GOLD stages	***P<0*.*001***	***P = 0*.*018***	***P<0*.*001***	***P = 0*.*039***
0	**-**	-	**1.85 (1.52; 2.25)**	**1.50 (1.02; 2.21)**
I	**1.61 (1.29; 2.00)**	**1.31 (1.05; 1.62)**	**1.58 (1.22; 2.06)**	**1.39 (1.08; 1.79)**
II	**1.59 (1.30; 1.95)**	**1.34 (1.09; 1.64)**	**1.54 (1.24; 1.90)**	**1.34 (1.07; 1.67)**
III-IV	1.00	1.00	1.00	1.00

* Simple sum of the number of self-reported comorbidities, considering the following: heart diseases, hypertension, diabetes, lung cancer, stroke (or embolism or ischemia), tuberculosis and gastritis (or ulcer).

**Fig 4:**
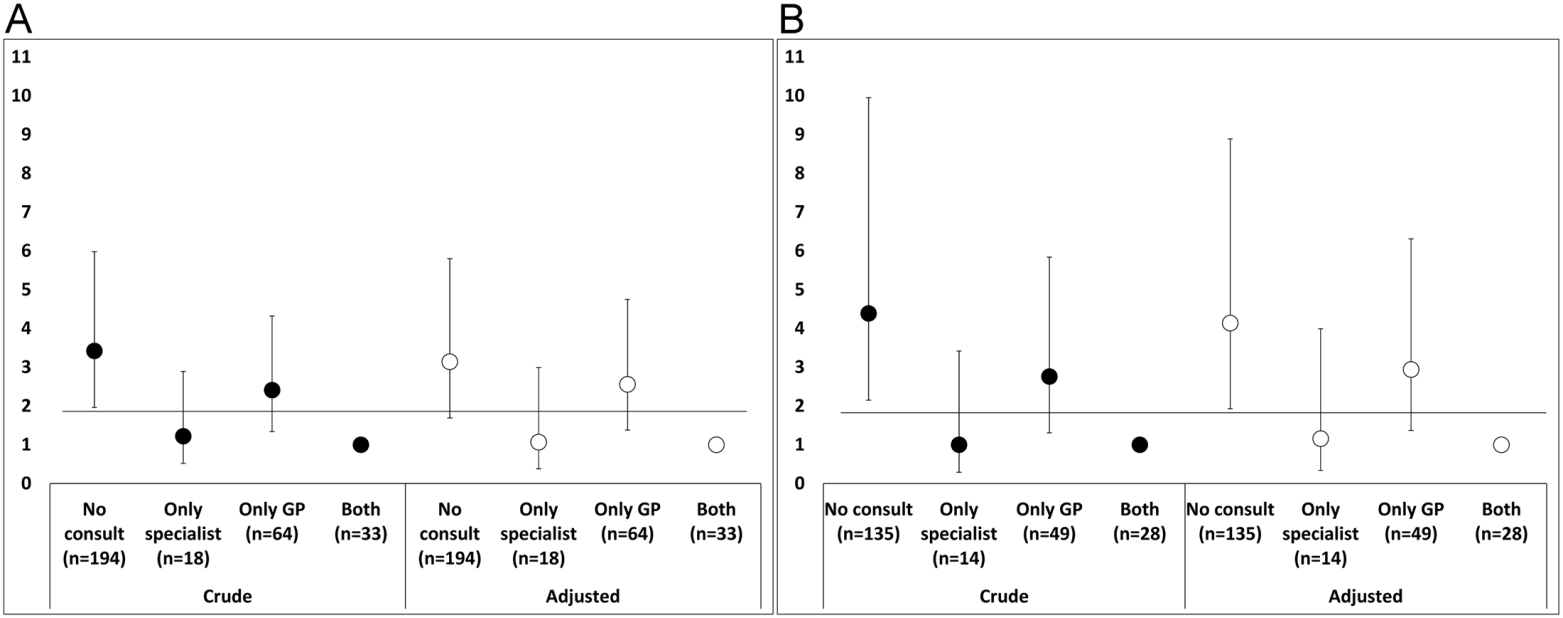
Prevalence ratios for underdiagnosis according to type of physician visited in past year, by COPD criteria: A) post-BD FEV_1_/FVC<0.70; and B) LLN.

Fig 4 shows the prevalence ratios for underdiagnosis according to the type of physician visited in the past year. According to both COPD criteria (A: post-BD FEV_1_/FVC <0.70, and B: LLN), those who did not visit a clinician in the last year or those who only attended the GP have more risk of underdiagnosis than those who attended “both” (GP and specialist).

The proportion of COPD misdiagnosis (26.4%) and underdiagnosis (65.8%) by post-BD FEV_1_/FVC <0.70 criteria decreased (S1 Fig) among those patients who had performed spirometry in the past 12 months (self reported).

## Discussion

The most important findings of this study of COPD underdiagnosis in the Latin American primary care setting were: first, using the post-BD FEV_1_/FVC <0.70 definition of COPD, the prevalence of COPD underdiagnosis was 77% and using the LLN definition was 73%. Second, COPD underdiagnosis was associated with higher BMI (≥30 kg/m^2^), milder airway obstruction (GOLD I–II), black color, absence of dyspnea, wheezing, and no history of exacerbations and hospitalizations due to exacerbations in the past year. Third, assistance by a specialist was protective for COPD underdiagnosis. Fourth, having performed spirometry in past 12 months decreased COPD underdiagnosis and misdiagnosis.

Several studies have assessed the prevalence of COPD underdiagnosis in different settings [1,5–7,12]. Recently, Lamprecht et al [1] analyzed the underdiagnosis of COPD and its determinants in national and international surveys of general populations (44 sites from 27 countries and 30,874 participants). They found that overall, 81.4% of COPD cases (defined as post-BD FEV_1_/FVC <LLN) were underdiagnosed; there was considerable variation across sites, and underdiagnosis was associated with male gender, younger age, never and current smoking, poor education, no previous spirometry and lower airflow limitation [1]. To our knowledge no previous study has evaluated the prevalence of underdiagnosis in COPD using different diagnostic spirometric criteria.

On the other hand, limited information exists regarding the prevalence of COPD underdiagnosis in a primary care setting [5–7]. Bednarek et al [5] reported an 11% prevalence of post-BD airflow limitation among 1,960 participants from a primary care population in Poland. Only 18.6% of these patients had previously been diagnosed with COPD (81.4% underdiagnosed). Similar findings have been reported by others [7]. In a population similar to the present study, Hill et al [6] measured the prevalence of COPD in an at-risk population who visited a primary care practitioner for any reason. Of the 1,003 participants, 208 met the spirometric criteria used for COPD (GOLD stage ≥2), and only 67/208 had self-reported diagnosis of COPD (68% underdiagnosed) [6]. The results of our investigation are consistent with those of epidemiological surveys of COPD and indicate that the disease is largely underdiagnosed and varies across the countries of the study; the magnitude of the problem appears to be especially great in Venezuela and Uruguay. As expected the overall COPD underdiagnosis prevalence using the post-BD FEV_1_/FVC <0.70 definition was higher (15.5%) compared to the LLN definition (10.7%). The wide variation in the prevalence of undiagnosed COPD across the regions studied is noteworthy (Fig 2). It is important to mention that the percentage of COPD underdiagnosis was not similar among the sites (Fig 1), and there was no good match between the COPD prevalence by spirometry criteria and the prevalence of underdiagnosis, so these suggest that the underlying prevalence of COPD is probably not a main determinant of undiagnosed COPD prevalence.

Our results imply that there are differences and problems in the primary care setting of some countries that may influence the incidence of COPD underdiagnosis. The lack of similar information from other multinational studies in the primary care setting makes it difficult to compare results with the PUMA study.

In population-based surveys, Lamprecht et al [1] found that the main determinants for COPD underdiagnosis worldwide were male gender (except in Spain), younger age, never and current smoking, lower level of education, absence of respiratory symptoms, lack of previous spirometry, and milder airway obstruction. Data from a primary care population showed that underdiagnosed COPD patients described fewer respiratory symptoms [6]. In the present study, we found that COPD underdiagnosis was associated with higher BMI (≥30 kg/m^2^), lower airway obstruction (GOLD I–II), black color, absence of dyspnea, wheezing, and no history of exacerbations and hospitalizations due to exacerbations in the past year. These results are in line with those reported in population based surveys, suggesting that patients with fewer respiratory symptoms and milder airway obstruction are less likely to receive a diagnosis.

Different studies have documented a widespread underuse of spirometry by general practitioners to establish COPD diagnosis [13–17]. De Miguel Díez et al [14] assessed the methods used by primary care physicians and chest physicians to diagnose COPD in Spain and analyzed the factors affecting the correct diagnosis of the disease. Their results indicated that primary care physicians classified 29.3% of the patients correctly, whereas chest physicians diagnosed 84.8% correctly. Spirometry was available to 49.1% of the primary care physicians and 97.8% of the chest physicians. Moreover, only 29.9% of the primary care settings had a technician in charge of performing the measurements, in comparison with 97.8% of the specialized chest physician settings [14]. De Miguel Díez et al [14] also indicated that the use of spirometry in diagnosing COPD was related to the level of patient care (primary or specialist) and the availability of the test in the primary care setting. Others have reported that the number of visits to a primary care physician for a respiratory problem was not a determining factor to avoid COPD underdiagnosis [5]. Our results support the this last finding and, in addition, indicate that those who did not asee a clinician in the last-year or only visited the GP have more risk of underdiagnosis and having performed spirometry in past 12 months decreases COPD underdiagnosis and misdiagnosis. All these findings clearly show that differences in diagnosis between levels of patient care are closely related to the availability of spirometry and the underuse of spirometry by primary care practitioners to establish a COPD diagnosis. The more restricted access of general practitioners to spirometry could explain, in part, the above results.

There are some limitations to the present study that deserve to be discussed. It is possible that the results found in this study do not apply to all Latin American countries. As a result of resources available, the sample size varied among centers and countries (from 104 eligible patients in Uruguay to 726 in Venezuela). Due to the smaller sample size in Uruguay and Colombia we recalculated the margin of error for prevalence for these two sites and they are: Uruguay (7.8% percentage points) and Colombia (4.35% percentage points). Uruguay is the worst scenario (considering an overall COPD prevalence of approximately 20% it means that the real prevalence in Uruguay could range from 12.2% to 27.8%). Nevertheless, even if a convenience sample was utilized in this study, the procedure used was the most reasonable in view of the operational possibilities in each country: to avoid selection bias, centers were selected on the basis of available lists of primary care physicians and the study patients were those who visited the center spontaneously. Another possible limitation is the fact that the study design did not include revision of the medical record, so the definition of prior diagnostic label of COPD was only determined using self-reported physician diagnosis of emphysema, chronic bronchitis, or COPD. This could overestimate the underdiagnosis rate of COPD; due to patients may unknown the exact diagnosis.

In summary, this study shows that irrespective of the spirometric definition used, COPD underdiagnosis is a major health problem in the primary care setting in Latin America, and highlights the need to improve the diagnosis of COPD in this setting. It is necessary to insist on the greater use and availability of spirometry in the primary care setting to enhance COPD diagnosis and to reduce misdiagnosis. It is also essential to optimize coordination between different levels of care to improve the disease approach.

## Supporting Information

S1 Fig Correct prior diagnosis, misdiagnosis and underdiagnosis (for COPD criteria FEV_1_/FVC <0.70 post-BD) among those who perform previous spirometry in past 12 months. (TIF)

S1 File PUMA questionnaire used during the study. (PDF)
