# Supplementary figures and images for: COPD Underdiagnosis and Misdiagnosis in a High-Risk Primary Care Population in Four Latin American Countries. A Key to Enhance Disease Diagnosis: The PUMA Study
Source: PLoS One. 2016 Apr 13;11(4):e0152266. doi: 10.1371/journal.pone.0152266 (PMC4830516; doi:10.1371/journal.pone.0152266)

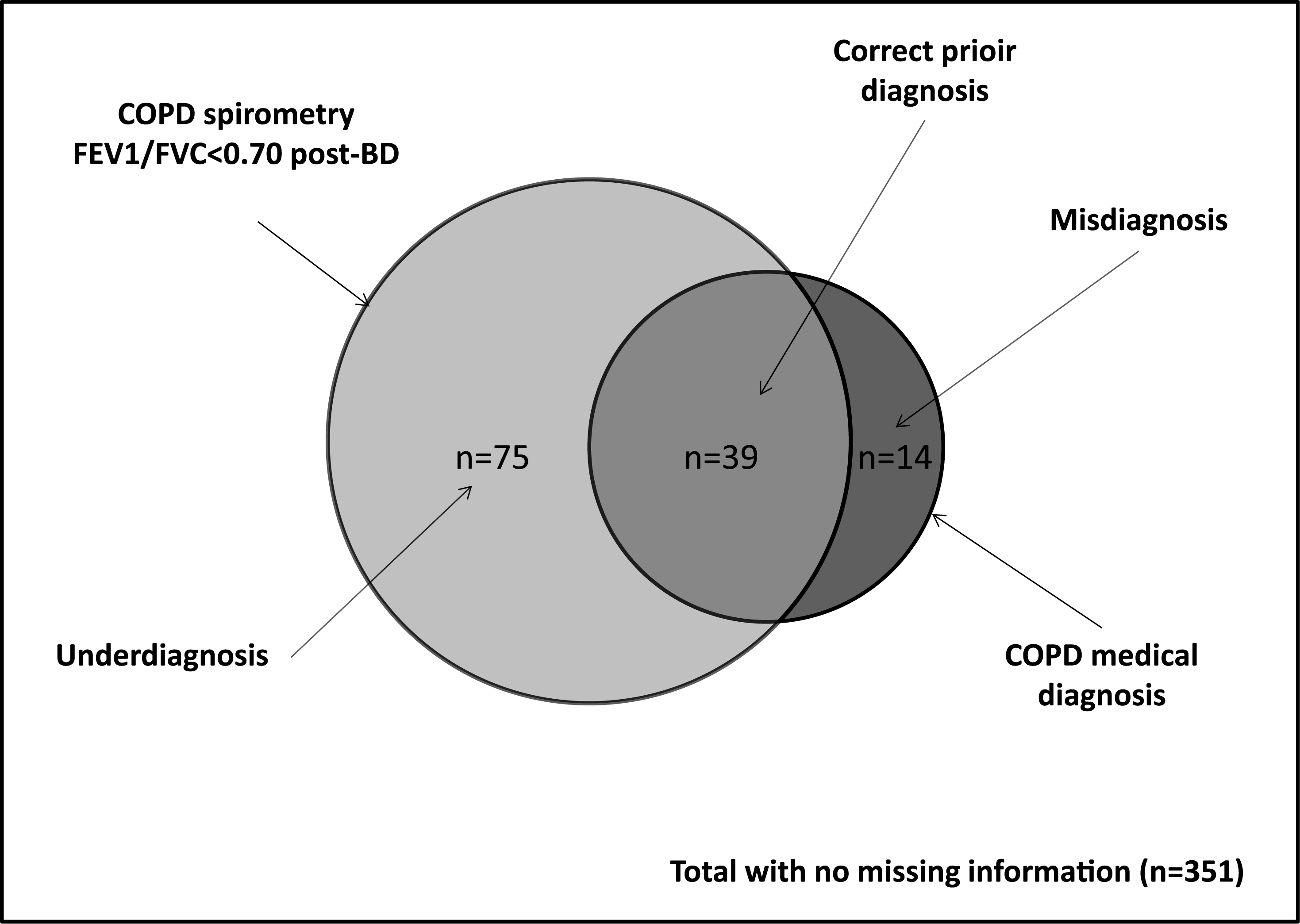

Supplement: S1 Fig — (TIF) [file pone.0152266.s001.tif]
