# Supplementary material for: COPD Underdiagnosis and Misdiagnosis in a High-Risk Primary Care Population in Four Latin American Countries. A Key to Enhance Disease Diagnosis: The PUMA Study
Source: PLoS One. 2016 Apr 13;11(4):e0152266. doi: 10.1371/journal.pone.0152266 (PMC4830516; doi:10.1371/journal.pone.0152266)
Supplement: S1 File — (PDF) [file pone.0152266.s002.pdf]

Site ID: ______________
Patient ID: ______________

PUMA QUESTIONNAIRE

DATE OF INTERVIEW:__ __ / __ __ / __ __ __ __
d d m m y y y y

INTERVIEW BEGAN AT: __ __ h __ __ min.
(24:00 hour format)

NAME OF THE INTERVIEWER:

____

Demographic Variables

1. SEX

1☐ male   2☐ female

2. WHAT IS YOUR RACE? (Read the options so the person interviewed can answer)

1☐ white
2☐ black
3☐ yellow (asian)
4☐ mixed race (*mestiza*)
5☐ indigenous

3. WHAT IS YOUR DATE OF BIRTH?

__ __ / __ __ / __ __ __ __
d d m m y y y y

4. HOW MANY FULL YEARS OF STUDY DO YOU HAVE?

_______ years

5. WHAT WAS THE LAST GRADE OF EDUCATION THAT YOU COMPLETED?

1☐ elementary
2☐ middle and high school
3☐ minor degree / other, non-university studies (Training College)
4☐ bachelor / Postgraduate / Master Degrees
5☐ none
6☐ does not know

ANTHROPOMETRY

Anthropometry

MEASURE THE INTERVIEWEE'S HEIGHT WITHOUT SHOES, SELECT THE UNITS OF MEASUREMENT ALONG WITH THE VALUE

6. HEIGHT OF THE PERSON INTERVIEWED:

___ ___ ___ , ___ ☐ cm ☐ in

7. WEIGHT OF THE PERSON INTERVIEWED:

___ ___ ___ , ___ ☐ Kg ☐ Lb

SPIROMETRY and OXIMETRY

QUESTIONS FOR EXCLUSION FROM UNDERGOING SPIROMETRY

8. HAVE YOU HAD SURGERY (AN OPERATION) OF THE LUNG, THORAX OR ABDOMEN, IN THE LAST 3 MONTHS?

1☐ yes   2☐ no

9. HAVE YOU HAD A HEART ATTACK OR MYOCARDIAL INFARCTION IN THE LAST 3 MONTHS?

1☐ yes   2☐ no

10. HAVE YOU HAD A RETINAL DETACHMENT OR EYE SURGERY (OPERATION) IN THE LAST 3 MONTHS?

1☐ yes   2☐ no

11. HAVE YOU BEEN HOSPITALISED FOR ANY OTHER HEART PROBLEM IN THE LAST 3 MONTHS?

1☐ yes   2☐ no

12. ARE YOU TAKING MEDICINE FOR TUBERCULOSIS AT THE MOMENT?

1☐ yes   2☐ no

13. ARE YOU PREGNANT AT THE MOMENT?

1☐ yes   2☐ no

Site ID: ______________
Patient ID: ______________

14. OXIMETRY

BEFORE TAKING MEASUREMENTS, MAKE SURE THAT BOTH THE PERSON INTERVIEWED AND THE TEAM MEET THE CRITERIA FOR PROPER MEASUREMENT-TAKING (SEE GUIDE)

MEASUREMENT NUMBER	PULSE (BPM)	OXYGEN SATURATION (%SaO_2)
1		
2		
3		
4		
5		
6		
	14A. Pulse (BPM)	14B. OXYGEN SATURATION (%SaO_2)
AVERAGE OF THE SIX MEASUREMENTS		

14C. WAS YOUR PULSE ABOVE 120 BPM?

1☐ yes 2☐ no

*If the person interviewed answered **"yes"** to any of the previous questions **THE SPIROMETRY MUST NOT BE PERFORMED, YOU MUST STOP THE INTERVIEW AND REFER THE PATIENT TO THE INVESTIGATOR INMEDIATLY**, the patient will be redirected in the study according to protocol, otherwise go to question 15*

QUESTIONS FOR ALL PERSONS INTERVIEWED WHO DO NOT MEET CRITERIA FOR EXCLUSION AND WHO THEREFORE MUST BE UNDERGO SPIROMETRY

15. HAVE YOU HAD A RESPIRATORY INFECTION (COLD) IN THE LAST 3 WEEKS?

1☐ yes 2☐ no

16. HAVE YOU TAKEN ANY DRUG OR MEDICINE FOR YOUR BREATHING (FOR YOUR LUNGS), SUCH AS AEROSOL SPRAYS, INHALANTS OR NEBULISERS, IN THE LAST 3 HOURS?

1☐ yes 2☐ no

17. HAVE YOU SMOKED ANY KIND OF CIGARETTE (CIGAR, PIPE) IN THE LAST TWO HOURS?

1☐ yes **¿How many?** ________ 2☐ no

18. HAVE YOU DONE ANY STRENUOUS PHYSICAL EXERCISE, SUCH AS GYM, WALKING OR JOGGING, IN THE LAST HOUR?

1☐ yes 2☐ no

19. TEST RESTULS:

1☐ Test complete

Test incomplete
(Mark one of the options below):

2☐ The person interviewed did not understand the instructions
3☐ The person interviewed was excluded for medical reasons (not eligible)
4☐ The person interviewed was not capable of undergoing the test (other reasons*, please specify on question 20)
5☐ The person interviewed rejected

20. * PLEASE TAKE NOTE OF ANYTHING ABOUT THE SPIROMETRY RELATED TO WHETHER OR NOT THE PERSON INTERVIEWED IS ABLE TO UNDERGO THE TEST (FOR INSTANCE: KYPHOSCOLIOSIS, DENTURE[S], MISSING LIMB[S], ETC).

....
....
....
....
....
....

RESPIRATORY SYMPTOMS AND DISEASES

NOW I AM GOING TO ASK YOU SOME QUESTIONS ABOUT YOUR BREATHING AND YOUR LUNGS. TRY TO ANSWER YES OR NO. IF IN DOUBT, JUST ANSWER NO.

Site ID: ______________
Patient ID: ______________

Coughing

21. DO YOU USUALLY COUGH WHITOUT HAVING A COLD?

1☐ yes 2☐ no

*[If **"yes"**, ask question 21A; if **"no"**, go to question 22]*

21A. ARE THERE MONTHS WHEN YOU COUGH MOST OF THE DAYS OR ALMOST EVERY DAY?

1☐ yes 2☐ no

*[If **"yes"**, ask question 21B and 21C; if **"no"**, go to question 22]*

21B. DO YOU COUGH MOST OF THE DAYS FOR AT LEAST THREE MONTHS A YEAR?

1☐ yes 2☐ no

21C. FOR HOW MANY YEARS HAVE YOU HAD THIS COUGH?

1☐ less than 2 years
2☐ between 2 and 5 years
3☐ more than 5 years

Phlegm

22. DO YOU USUALLY HAVE PHLEGM WHITOUT HAVING A COLD THAT COMES FROM YOUR LUNG OR PHLEGM THAT IS HARD TO GET RID OFF?

1☐ yes 2☐ no

*[If **"yes"**, go to question 22A; if **"no"**, go to question 23]*

22A. ARE THERE MONTHS ON WHICH YOU HAVE PHLEGM MOST OF THE DAYS OR ALMOST EVERY DAY?

1☐ yes 2☐ no

*[If **"yes"**, go to question 22B and 22C; if **"no"**, go to question 23]*

22B. DO YOU HAVE THESE PHLEGM MOST OF THE DAYS AT LEAST FOR THREE MONTHS A YEAR?

1☐ yes 2☐ no

22C. FOR HOW MANY YEARS HAVE YOU HAD THESE PHLEGM?

1☐ less than 2 years
2☐ between 2 and 5 years
3☐ more than 5 years

Whistling / Squeaking / Wheezing in chest

23. HAVE YOU HAD ANY WHEEZING / WHISTLING / SQUEAKING IN YOUR CHEST IN THE LAST 12 MONTHS?

1☐ yes 2☐ no

*[If **"yes"**, go to questions 23A and 23B; if **"no"**, go to question 24]*

23A. DID YOU ONLY HAVE THIS (WHEEZE / WHISTLE / SQUEAK IN YOUR CHEST WHEN YOU HAD A COLD IN THE LAST 12 MONTHS?

1☐ yes 2☐ no

23B. DID YOU EVER HAVE AN ATTACK OF WHEEZING / SQUEAKING / WHISTLING IN YOUR CHEST ALONG WITH DIFFICULTY BREATHING IN THE LAST 12 MONTHS?

1☐ yes 2☐ no

Shortness of Breath

24. DO YOU HAVE ANY PROBLEM THAT PREVENTS YOU FROM MOVING OR WALKING, THAT IS NOT DUE TO A LUNG OR HEART PROBLEM?

1☐ yes 2☐ no

*[Si **"yes"**, please ask, and write down, what problem(s) and then go to question 26; if **"no"**, go to question 25, and ask questions 25A, 25B, 25C and 25D, regardless of whether the answer to each of those questions were **yes** or **no**]*

WHAT PROBLEMS?...

....

....

Site ID: ______________
Patient ID: ______________

25. DO YOU FEEL TO HAVING DIFFICULTY BREATHING WHEN WALKING AT A FASTER PACE ON A FLAT PATH OR ON A SLIGHT SLOPE?

1☐ yes 2☐ no

25A. DO YOU HAVE TO WALK SLOWER ON A FLAT PATH THAN PEOPLE OF YOUR AGE BECAUSE YOU HAVE SHORTNESS OD BREATH?

1☐ yes 2☐ no

25B. DO YOU HAVE TO CATH YOUR BREATH WHEN WALKING ON THE FLAT AT YOUR NORMAL PACE?

1☐ yes 2☐ no

25C. DO YOU HAVE TO CATH YOUR BREATH WHEN WALKING ON THE FLAT AFTER WALKING ABOUT 100 METRES OR WHEN WALKING FROM ONE STREET CORNER TO THE NEXT?

1☐ yes 2☐ no

25D. IS YOUR SHORTNESS OF BREATH SO STRONG THAT YOU CANNOT LEAVE THE HOUSE OR EVEN GET DRESS?

1☐ yes 2☐ no

26. HAS A DOCTOR EVER TOLD YOU THAT YOU HAVE LUNGS EMPHYSEMA?

1☐ yes 2☐ no

27. HAVE YOU EVER IN YOUR LIFE BEEN TOLD BY A DOCTOR THAT YOU HAVE ASTHMA, ASTHMATIC BRONCHITIS, BRONCHOSPASM OR ALLERGIC BRONCHITIS?

1☐ yes 2☐ no

*[If "**yes**", continue to 27A; If "**no**", go to question 28]*

27A. ARE YOU CURRENTLY SUFFERING FROM ASTHMA, BRONCHITIS, ASTHMATIC BRONCHITIS OR BRONCHOSPASM OR ALLERGIC BRONCHITIS?

1☐ yes 2☐ no

28. HAVE YOU EVER IN YOUR LIFE BEEN TOLD BY A DOCTOR THAT YOU HAVE CHRONIC BRONCHITIS?

1☐ **yes** 2☐ **no**

*[If "**yes**", ask 28A; If "**no**", go to question 29]*

28A. ARE YOU STILL SUFFERING FROM CHRONIC BRONCHITIS?

1☐ yes 2☐ no

29. HAVE YOU EVER IN YOUR LIFE BEEN TOLD BY A DOCTOR THAT YOU HAVE CHRONIC OBSTRUCTIVE PULMONARY DISEASE (COPD)?

1☐ yes 2☐ no

<u>Management</u>

NOW I AM GOING TO ASK YOU ABOUT ANY MEDICINE YOU MAY BE TAKING TO HELP YOUR BREATHING OR YOUR LUNGS.

I WOULD LIKE TO KNOW ABOUT ANY MEDICATION YOU ARE USING ON A REGULAR BASIS (CONSTANTLY) AND ALSO ABOUT THE ANY MEDICATION YOU ONLY USE WHEN YOU ARE NOT FEELING WELL.

I WOULD ALSO LIKE YOU TO TELL ME ABOUT EACH ITEM OF MEDICATION MEDICINE YOU TAKE, HOW YOU TAKE IT AND HOW MANY TIMES A MONTH YOU TAKE IT.

30. <u>IN THE LAST 12 MONTHS</u>, DID YOU TOOK ANY MEDICATION FOR YOUR LUNGS OR YOUR BREATHING?

1☐ yes 2☐ no

*[If **"yes"**, go to question 30A, if it is **"no"** go to question 31]*

**30A. MEDICATION NAME**					
**30B. MEDICATION CODE**					
**30C. FORMULATION**	1☐ tablets<br>2☐ inhaler<br>3☐ nebuliser<br>4☐ syrup<br>5☐ suppository<br>6☐ injection<br>7☐ Other	1☐ tablets<br>2☐ inhaler<br>3☐ nebuliser<br>4☐ syrup<br>5☐ suppository<br>6☐ injection<br>7☐ Other	1☐ tablets<br>2☐ inhaler<br>3☐ nebuliser<br>4☐ syrup<br>5☐ suppository<br>6☐ injection<br>7☐ Other	1☐ tablets<br>2☐ inhaler<br>3☐ nebuliser.<br>4☐ syrup<br>5☐ suppository<br>6☐ injection<br>7☐ Other	1☐ tablets<br>2☐ inhaler<br>3☐ nebuliser<br>4☐ syrup<br>5☐ suppository<br>6☐ injection<br>7☐ Other
**30D. DO YOU TAKE THAT MEDICATION: MOST OF THE DAYS; ONLY WHEN YOU FEEL SYMPTOMS OR DISCOMFORT; OR IN BOTH CASES (ALWAYS)?**	1☐ most days<br>2☐ symptoms<br>3☐ both (always)<br>*[If the answer is **"most days"** only ask 30E; if it is **'symptoms'** or **'both'** ask 30E and 30F]*	1☐ most days<br>2☐ symptoms<br>3☐ both (always)<br>*[If the answer is **"most days"** only ask 30E; if it is **'symptoms'** or **'both'** ask 30E and 30F]*	1☐ most days<br>2☐ symptoms<br>3☐ both (always)<br>*[If the answer is **"most days"** only ask 30E; if it is **'symptoms'** or **'both'** ask 30E and 30F]*	1☐ most days<br>2☐ symptoms<br>3☐ both (always)<br>*[If the answer is **"most days"** only ask 30E; if it is **'symptoms'** or **'both'** ask 30E and 30F]*	1☐ most days<br>2☐ symptoms<br>3☐ both (always)<br>*[If the answer is **"most days"** only ask 30E; if it is **'symptoms'** or **'both'** ask 30E and 30F]*
**30E. WHEN YOU TAKE THAT MEDICATION, HOW MANY DAYS A WEEK DO YOU TAKE IT?**	____ days	____ days	____ days	____ days	____ days
**30F. WHEN DID YOU USE THAT MEDICATION IN THE LAST 12 MONTHS, FOR HOW MANY MONTHS DID YOU TAKE IT?**	1☐ 0-3 months<br>2☐ 4-6 months<br>3☐ 7-9 months<br>4☐ 10-12 months	1☐ 0-3 months<br>2☐ 4-6 months<br>3☐ 7-9 months<br>4☐ 10-12 months	1☐ 0-3 months<br>2☐ 4-6 months<br>3☐ 7-9 months<br>4☐ 10-12 months	1☐ 0-3 months<br>2☐ 4-6 months<br>3☐ 7-9 months<br>4☐ 10-12 months	1☐ 0-3 months<br>2☐ 4-6 months<br>3☐ 7-9 months<br>4☐ 10-12 months

Site ID: ______________
Patient ID: ______________

**30A. MEDICATION NAME**					
**30B. MEDICATION CODE**					
**30C. FORMULATION**	1☐ tablets<br>2☐ inhaler<br>3☐ nebuliser<br>4☐ syrup<br>5☐ suppository<br>6☐ injection<br>7☐ Other	1☐ tablets<br>2☐ inhaler<br>3☐ nebuliser<br>4☐ syrup<br>5☐ suppository<br>6☐ injection<br>7☐ Other	1☐ tablets<br>2☐ inhaler<br>3☐ nebuliser<br>4☐ syrup<br>5☐ suppository<br>6☐ injection<br>7☐ Other	1☐ tablets<br>2☐ inhaler<br>3☐ nebuliser.<br>4☐ syrup<br>5☐ suppository<br>6☐ injection<br>7☐ Other	1☐ tablets<br>2☐ inhaler<br>3☐ nebuliser<br>4☐ syrup<br>5☐ suppository<br>6☐ injection<br>7☐ Other
**30D. DO YOU TAKE THAT MEDICATION: MOST OF THE DAYS; ONLY WHEN YOU FEEL SYMPTOMS OR DISCOMFORT; OR IN BOTH CASES (ALWAYS)?**	1☐ most days<br>2☐ symptoms<br>3☐ both (always)<br>*[If the answer is **"most days"** only ask 30E; if it is **'symptoms'** or **'both'** ask 30E and 30F]*	1☐ most days<br>2☐ symptoms<br>3☐ both (always)<br>*[If the answer is **"most days"** only ask 30E; if it is **'symptoms'** or **'both'** ask 30E and 30F]*	1☐ most days<br>2☐ symptoms<br>3☐ both (always)<br>*[If the answer is **"most days"** only ask 30E; if it is **'symptoms'** or **'both'** ask 30E and 30F]*	1☐ most days<br>2☐ symptoms<br>3☐ both (always)<br>*[If the answer is **"most days"** only ask 30E; if it is **'symptoms'** or **'both'** ask 30E and 30F]*	1☐ most days<br>2☐ symptoms<br>3☐ both (always)<br>*[If the answer is **"most days"** only ask 30E; if it is **'symptoms'** or **'both'** ask 30E and 30F]*
**30E. WHEN YOU TAKE THAT MEDICATION, HOW MANY DAYS A WEEK DO YOU TAKE IT?**	____ days	____ days	____ days	____ days	____ days
**30F. WHEN DID YOU USE THAT MEDICATION IN THE LAST 12 MONTHS, FOR HOW MANY MONTHS DID YOU TAKE IT?**	1☐ 0-3 months<br>2☐ 4-6 months<br>3☐ 7-9 months<br>4☐ 10-12 months	1☐ 0-3 months<br>2☐ 4-6 months<br>3☐ 7-9 months<br>4☐ 10-12 months	1☐ 0-3 months<br>2☐ 4-6 months<br>3☐ 7-9 months<br>4☐ 10-12 months	1☐ 0-3 months<br>2☐ 4-6 months<br>3☐ 7-9 months<br>4☐ 10-12 months	1☐ 0-3 months<br>2☐ 4-6 months<br>3☐ 7-9 months<br>4☐ 10-12 months

31. PLEASE TELL ME ABOUT ANYTHING ELSE YOU HAVEN'T MENTIONED SO FAR THAT YOU ARE USING OR DOING TO HELP YOUR BREATHING OR YOUR LUNGS, FOR EXAMPLE: HOMEOPATHY, HERBAL PREPARATIONS, VITAMINS, BREATHING EXERCISES, PHYSIOTHERAPY, SWIMMING, ACUPUNCTURE, MASSAGE, ANY KIND OF SPECIAL FOOD, REHABILITATION, ETC.

Therapies, Remedies or any other activity	Code

32. HAS A DOCTOR OR OTHER HEALTHCARE PROFESSIONAL IN YOUR LIFE ASKED YOU TO BLOW INTO SOME KIND OF DEVICE (CALLED A SPIROMETER OR PEAK FLOW METER) TO KNOW ABOUT THE FUNCTIONING OF YOUR LUNGS?

1☐ yes 2☐ no

*[If "**yes**", ask 32A; If "**no**", go to question 33]*

32A. HAVE YOU USED THAT DEVICE <u>IN THE LAST 12 MONTHS</u>?

1☐ yes 2☐ no

<u>**COPD Ambulatory Medical control**</u>

IN THE LAST 12 MONTHS:

33. HOW MANY TIMES HAVE YOU HAD TO CONSULT WITH

33A. GENERAL PRACTITIONER OR FAMILY DOCTOR?

____times

33B. AN SPECIALIST?

____times

<u>**Exacerbations/ Aggravations**</u>

IN THE LAST 12 MONTHS:

34. HAVE YOU HAD AN ACUTE CHANGE IN YOUR USUAL BREATHING SYMPTOMS, (SHORTNESS OF BREATH AND/OR COUGHING AND/OR PHLEGM) DIFFERENT THAN HOW IT USUALLY VARIES DAILY, THAT LEAD YOU TO CHANGE YOUR USUAL MEDICATION?

1☐ yes 2☐ no

*[If "**yes**" go to 34A, 34B and 34C. If "**no**" go to question 35]*

34A. HOW MANY TIMES HAVE YOU HAD THESE ACUTE CHANGES IN YOUR USUAL BREATHING SYMPTOMS?

____times

IN THE LAST 12 MONTHS:

34B. ABOUT THESE CHANGES, HOW MANY TIMES HAVE YOU HAD TO CONSULT WITH..

34B1. GENERAL PRACTITIONER OR FAMILY DOCTOR?

____times

34B2. AN SPECIALIST?

____times

34B3. AN EMERGENCY SPECIALIST?

____times

IN THE LAST 12 MONTHS:

34C. HOW MANY TIMES HAVE YOU HAD TO BE HOSPITALISED BECAUSE OF THESE CHANGES?

____times

34C1. HOW MANY DAYS, ON AVERAGE, HAVE YOU HAD TO BE HOSPITALISED BECAUSE OF THESE CHANGES?

____times

34C2. HAVE YOU HAD TO BE ADMITTED IN AN INTENSIVE CARE UNIT BECAUSE OF THESE CHANGES?

1☐ yes, Specify N° times: ____ 2☐ no

[Ask question 35 for all the participants]

35. HAS THERE EVER BEEN A PERIOD IN YOUR LIFE WHEN YOUR BREATHING (LUNG) PROBLEMS WERE SO STRONG THAT THEY PREVENTED YOU TO CARRYING OUT YOUR DAILY ACTIVITIES OR PREVENTED YOU FROM GOING TO WORK?

1☐ yes 2☐ no

*[If "**yes**", ask 35A; If "**no**", go to question 36]*

35A. HOW MANY TIMES DO YOU FEEL IN THAT WAY IN THE LAST 12 MONTHS?

_____ times

*[If question 35A is **>0** go to 35B; if question 35A is **= 0** then go to 36]*

35B. HOW MANY TIMES DID YOU NEED TO SEE THE DOCTOR FOR THIS PROBLEM IN THE LAST 12 MONTHS?

_____ times

*[Even if it's **"0"** times, ask question 35C]*

35C. HOW MANY TIMES HAVE YOU NEEDED TO BE HOSPITALISED FOR THIS PROBLEM IN THE LAST 12 MONTHS?

_____ times

*[If question 35C is **>0** go to 35C1; if question 35C is **=0** go to 36]*

35C1. HOW MANY DAYS IN ALL HAVE YOU BEEN HOSPITALISED FOR LUNG PROBLEMS IN THE LAST 12 MONTHS?

_____ days

Smoking

NOW I AM GOING TO ASK YOU ABOUT YOUR SMOKING HABITS. FIRST OF ALL I AM GOING TO ASK YOU ABOUT CIGARRETTE SMOKING.

36. HAVE YOU EVER IN YOUR LIFE SMOKED CIGARETTES?

1☐ yes 2☐ no

*[If the person interviewed smoked less than 20 packs in their whole life or less than 1 cigarette a day in one year, code as **"no".]***
*[If "**yes**", ask questions 36A to 36C; if **"no"**, go to question 38]*

36A. HOW OLD WERE YOU WHEN YOU STARTED SMOKING ON A REGULAR BASIS?

_____ years

*[**"Regular basis"** means at least 1 cigarette every 30 days]*

36B. HOW OLD WERE YOU WHEN YOU GAVE UP SMOKING CIGARETTES COMPLETELY?

_____ years

36C. WHEN YOU DID SMOKE, HOW MANY CIGARETTES ON AVERAGE DID YOU SMOKE A DAY?

_____ cigarettes/ day

37. DO YOU CURRENTLY SMOKE ANY TYPE OF CIGARETTES?

1☐ yes 2☐ no

*[**"Currently"** means any cigarettes at all in the last 30 days. If "**no**" go to question 38; If "**yes**" continue with questions 37A to 37B]*

37A. HOW MANY CIGARETTES A DAY DO YOU SMOKE?

_____ cigarettes/ day

37B. HOW OLD WHERE YOU WHEN YOU BECAME A REGULAR SMOKER?

_____ years

*[**"Regular smoker"** means at least 1 cigarette every 30 days]*

37C. DURING ALL THE TIME YOU SMOKED, HOW MANY CIGARETTES A DAY, ON AVERAGE, DID YOU USED TO SMOKE A DAY?

_____ cigarettes/ day

38. HAVE YOU EVER IN YOUR LIFE DID SMOKE A PIPE OR CIGARS?

1☐ yes 2☐ no

38A. DO YOU CURRENTLY SMOKE A PIPE OR CIGARS?

1☐ yes 2☐ no

__"Currently"__ means 50 or more pipes and/or cigars lit up in the last 30 days.
*[If the person interviewed has never smoked (i.e. answered **"no"** to questions 36, 37, 38 and 38A), just go to question 42]*
*[If the person interviewed has at any time smoked (i.e. answered **"yes"** to any of questions 36 to 38A), keep going and ask question 42]*

39. HAS A DOCTOR EVER IN YOUR LIFE ADVISED YOU TO GIVE UP SMOKING?

1☐ yes 2☐ no

*[If "**yes**" ask: 39A to current smokers only and 39B to everyone, if "**no**" go straight to question 40]*

39A. HAVE YOU BEEN ADVISED TO GIVE UP SMOKING IN THE LAST 12 MONTHS?

1☐ yes 2☐ no

39B. HAVE YOU EVER USED ANY KIND OF TREATMENT PRESCRIBED TO YOU BY A DOCTOR TO HELP YOU GIVE UP SMOKING?

1☐ yes 2☐ no

*[If "**yes**", ask 39B1 and then question 40; if "**no**", go to question 40]*

39B1. WHAT TYPE OF MEDICATION DID YOU USE TO HELP YOU GIVE UP SMOKING?

1☐ Nicotine substitute (chewing gum, patches, aerosols)
2☐ Bupropion
3☐ Varenicline
9☐ Others (tofranil, etc.)

Others Specify..

40. HAVE YOU EVER USED ANYTHING NOT PRESCRIBED, TO HELP YOU STOP SMOKING?

1☐ yes 2☐ no

41. HAVE YOU EVER USED OR DONE ANYTHING ELSE TO HELP YOU STOP SMOKING?

1☐ yes 2☐ no

*[If "**yes**" ask 41A; otherwise go to question 42]*

41A. WHAT DID YOU DO?

1☐ hypnosis
2☐ acupuncture
3☐ others (laser, etc.)

Occupational exposure

42. HAVE YOU EVER IN YOUR LIFE DID WORK FOR ONE OR MORE YEARS IN A JOB WHERE THERE WERE DUSTS, SMOKE OR FUMES?

1☐ yes 2☐ no

*[If "**yes**" ask 42A; otherwise go to question 43]*

42A. FOR HOW MANY YEARS DID YOU WORK IN PLACES LIKE THAT?

________years

Other diseases

43. HAVE YOU EVER IN YOUR LIFE BEEN TOLD BY A DOCTOR THAT YOU HAD OR HAVE ANY OF THE FOLLOWING DISEASES:

43A. HEART DISEASES?

1☐ yes 2☐ no

43B. HIGH BLOOD PRESSURE (HYPERTENSION)?

1☐ yes 2☐ no

*[If 43B is "**yes**" then ask 43B1; if "**no**" go to question 43C]*

43B1. ARE YOU CURRENTLY TAKING ANY MEDICATION TO CONTROL YOUR BLOOD PRESSURE?

1☐ yes **2**☐ no

43C. DIABETES (HIGH BLOOD SUGAR)?

1☐ yes 2☐ no

43D. LUNG CANCER?

1☐ yes 2☐ no

43E. STROKE, EMBOLISM, ISCHEMIA?

1☐ yes 2☐ no

43F. TUBERCULOSIS?

1☐ yes 2☐ no

*[If 43F is "**yes**" then ask 43F1; otherwise go to question 43G]*

43F1. HAVE YOU EVER TOOK ANY TREATMENT FOR TUBERCULOSIS?

1☐ yes 2☐ no

43G. GASTRITIS OR ULCER?

1☐ yes 2☐ no

44. *IN THE LAST 12 MONTHS,* HOW MANY TIMES HAVE YOU HAD TO BE HOSPITALISED BECAUSE OF THESE OTHER DISEASES?

______ times

45. HAVE YOU EVER IN YOUR LIFE HAD AN OPERATION (SURGERY) IN WHICH A PART OF YOUR LUNG WAS REMOVED?

1☐ yes 2☐ no

46. WERE YOU HOSPITALISED IN YOUR CHILHOOD (WHEN YOU WERE UNDER 10 YEARS OF AGE) FOR LUNG PROBLEMS?

1☐ yes 2☐ no

47. HAVE YOU HAD A FLU VACCINATION IN THE LAST 12 MONTHS?

1☐ yes 2☐ no

48. HAS ANY DOCTOR OR HEALTHCARE PROFESSIONAL EVER TOLD YOU THAT YOUR FATHER, MOTHER, BROTHERS OR SISTERS WERE DIAGNOSED FOR CHRONIC EMPHYSEMA, CHRONIC BRONCHITIS OR COPD?

1☐ yes 2☐ no

49. HAVE YOU BEEN LIVING WITH ANYONE WHO SMOKES ANY KIND OF CIGARETTE, PIPE OR CIGAR IN YOUR HOUSE WITHIN THE LAST TWO WEEKS?

1☐ yes 2☐ no

NOW I AM GOING TO ASK YOU SOME QUESTIONS ABOUT OTHER EXPOSURES TO TOBACCO SMOKE, BUT IN THE LAST 12 MONTHS

49A. NOT COUNTING YOURSELF, HOW MANY PEOPLE USUALLY SMOKE IN YOUR HOME?

0☐ none ____ people

*[If the answer to question 49A was **"none"**, go to question 49C, otherwise answer 49B and continue]*

49B. FOR HOW MANY HOURS EACH DAY ARE YOU EXPOSED TO OTHER PEOPLE'S CIGARETTE SMOKE AT HOME?

0☐ none ____ hours

49C. FOR HOW MANY HOURS A DAY ARE YOU EXPOSED TO OTHER PEOPLE'S CIGARETTE SMOKE IN OTHER PLACES?

0☐ none ____ hours

49D. DID YOU FATHER REGULARY SMOKE, DURING YOUR CHILDHOOD?

1☐ yes
2☐ no
9☐ don't know

49E. DID YOU MOTHER REGULARLY SMOKE DURING YOUR CHILDHOOD OR BEFORE YOU WERE BORN?

1☐ yes
2☐ no
3☐ don't know

*[If "**yes**" go to question 49F, if "**no**" go to question 50]*

49F. WHEN YOUR MOTHER WAS PREGNANT WITH YOU:

1☐ she stopped smoking before getting pregnant
2☐ she diminished or gave up smoking during the pregnancy
3☐ she smoked as usually during the pregnancy
4☐ don't know

Indoor air pollution

50. WAS A COAL BURNING STOVE USED IN YOUR HOME FOR COOKING FOR MORE THAN 6 MONTHS OF YOUR LIFE?

1☐ yes 2☐ no

*[If "**yes**", go on to questions 50A to 50D; otherwise, go to question 51]*

50A. FOR HOW MANY YEARS WAS A <u>COAL BURNING STOVE USED FOR COOKING IN YOUR HOME?</u>

___ years

50B. HOW MANY HOURS A DAY ON AVERAGE DID YOU SPEND NEAR BY THE COAL <u>BURNING STOVE</u>?

___ hours

50C. IS A <u>COAL BURNING STOVE</u> STILL USED TO COOK IN YOUR HOME?

1☐ yes 2☐ no

50D. DOES THIS STOVE HAVE OR DID HAVE A CHIMNEY?

1☐ yes 2☐ no

51. WERE <u>WOOD, FIREWOOD, MANURE, CROP RESIDEUES, STRAW OR LEAVES</u> USED IN YOUR HOME FOR COOKING FOR MORE THAN 6 MONTHS OF YOUR LIFE?

1☐ yes 2☐ no

*[If "**yes**", go to questions 51A to 51D; otherwise go to question 52]*

51A. FOR HOW MANY YEARS WERE <u>WOOD, FIREWOOD, MANURE, CROP RESIDUES, STRAW OR LEAVES</u> USED FOR COOKING IN YOUR HOME?

____ years

51B. ON AVERAGE, HOW MANY HOURS A DAY DID YOU SPEND NEAR BY THE FIRE OF THE STOVE FUELLED BY <u>WOOD, FIREWOOD, MANURE, CROP RESIDUES, STRAW OR LEAVES?</u>

____ hours

51C. DO YOU STILL USE <u>WOOD, FIREWOOD, MANURE, CROP RESIDUE, STRAW OR LEAVES</u> TO COOK IN YOUR HOME?

1☐ yes 2☐ no

51D. DOES THIS STOVE HAVE A CHIMNEY?

1☐ yes 2☐ no

52. HAVE YOU USED COAL TO HEAT YOUR HOME FOR MORE THAN 6 MONTHS?

1☐ yes 2☐ no

*[If "**yes**", go to questions 52A to 52C; otherwise go to question 53]*

52A. FOR HOW MANY YEARS HAVE YOU USED <u>COAL</u> TO HEAT YOUR HOME?

___ years

52B. DO YOU STILL USE <u>COAL</u> TO HEAT YOUR HOME?

1☐ yes 2☐ no

52C. HOW MANY DAYS A YEAR ON AVERAGE A YEAR DID YOU SPEND NEAR BYTHAT HEATER?

___ days

53. WERE <u>WOOD, FIREWOOD, MANURE, CROP RESIDUES OR LEAVES</u> USED TO HEAT YOUR HOME FOR MORE THAN 6 MONTHS OF YOUR LIFE?

1☐ yes 2☐ no

*[If "**yes**", go to questions 53A to 53C; if "**no**" go to question 54]*

53A. FOR HOW MANY YEARS WAS <u>WOOD, FIREWOOD, MANURE, CROP RESIDUES, STRAW OR LEAVES</u> USED TO HEAT YOUR HOME?

____ years

53B. ARE <u>WOOD, FIREWOOD, MANURE, CROP RESIDUES, STRAW OR LEAVES</u> STILL USED TO HEAT YOUR HOME?

1☐ yes 2☐ no

53C. HOW MANY DAYS ON AVERAGE A YEAR DID YOU SPEND NEAR BY THAT HEATER?

____days

Use of Oxygen

54. DO YOU CURRENTLY USE OXYGEN AT HOME AS TREATMENT?

1☐ yes   2☐ no

*[If "**yes**", ask questions 54A and 54B; if "**no**" go to question 55]*

54A. HOW MANY HOURS A DAY DO YOU USE THE OXYGEN?

_____ hours

54B. DO YOU PAY FOR THE OXYGEN YOU USE OR IS IT PROVIDED BY AN INSTITUTION?

1☐ Paid for   2☐ Provided

Medical Service

55. ARE YOU ENTITLED TO ANY HEALTHCARE SERVICES IN AN INSTITUTION?

1☐ yes   2☐ no

*[If "**yes**" ask 55A; otherwise say thank you and fill in the end-of-interview data]*

55A. WHAT KIND OF INSTITUTION ARE YOU ENTITLED TO HEALTHCARE? (Mark all applicable)

1☐ PUBLIC
2☐ PRIVATE HEALTH INSURANCE
3☐ COMMUNITY-BASED HEALTH PLAN
4☐ OTHERS

55B. DOES YOUR HEALTHCARE SERVICES PAID FOR YOU LUNG MEDICATION? (Mark all applicable)

1☐ YES
2☐ NO
3☐ PARTIALLY

TIME INTERVIEW ENDED: ___ ___ h ___ ___ min.
(24:00 hour format)
